# Triangular osteosynthesis and lumbopelvic fixation as a valid surgical treatment in posterior pelvic ring lesions: a systematic review

**DOI:** 10.3389/fsurg.2024.1266393

**Published:** 2024-02-22

**Authors:** Giuseppe Rovere, Domenico De Mauro, Amarildo Smakaj, Giulia Sassara, Rocco De Vitis, Pasquale Farsetti, Lawrence Camarda, Giulio Maccauro, Francesco Liuzza

**Affiliations:** ^1^ Department of Orthopaedics and Traumatology, Fondazione Policlinico Universitario A. Gemelli IRCCS-Università Cattolica del Sacro Cuore, Rome, Italy; ^2^ Department of Clinical Science and Translational Medicine, Section of Orthopaedics and Traumatology, University of Rome “Tor Vergata”, Rome, Italy; ^3^ Department of Orthopaedic Surgery (DICHIRONS), University of Palermo, Palermo, Italy

**Keywords:** lumbopelvic fixation, triangular fixation, pelvis, internal fixators, infix, osteosynthesis

## Abstract

**Objective:**

Unstable fractures of the sacrum often occur in patients with pelvic fractures and represent a real challenge for the orthopedic surgeon. Triangular osteosynthesis (TOS) and lumbopelvic fixation (LP) may represent a valid management option for the treatment of this condition. We present a systematic literature review about lumbopelvic fixation and triangular fixation as treatment option for unstable sacral fractures, to assess clinical and radiological outcomes after surgery and to evaluate appropriate indications and impact on the natural history of sacral fractures.

**Methods:**

The review is reported according to the Preferred Reporting Items for Systematic Reviews and Meta-Analyses (PRISMA) guidelines. 50 articles out of 108 titles, were considered eligible for the full-text analysis. Finally, 16 studies that met inclusion criteria were included in this review.

**Results:**

Overall, 212 patients (87 males, 58 females) with sacral fractures treated with TOS triangular fixation or LP lumbopelvic fixation were collected. The mean age was 37.6 years. Mean follow-up reported in all studies was 24.14 months.

**Conclusion:**

The results presented by the different authors, highlight the effectiveness of TOS triangular fixation and LP lumbopelvic fixation for the treatment of unstable sacral fractures associated with other pelvic fractures, in terms of function, stability, cost-effectiveness, and quality of life postoperatively.

## Background

Unstable fractures of the sacrum often occur in patients with pelvic fractures (1), determining a real challenge for orthopedic surgeons, due to high rates of secondary dislocation (up to 15%), mostly caused by lesions with an associated vertical instability (2). Among usual surgical treatment taken into account facing those fractures, there are iliosacral screw fixation and posterior plate as tension band osteosynthesis, but none of them can adequately prevent potential vertical displacement (3). To better deal with vertical instability, Käch and Trentz (4) in 1,994 proposed for the first time the lumbopelvic fixation as surgical option in pelvic fractures involving the sacrum, specifically those lesions described as Vertical Shear according to Young and Burgess classification (5). The surgical technique was then developed and improved during the years, undergoing a deep revisitation through the original idea from Schildauer et al. to add to the lumbopelvic fixation an ileo-sacral screw, in the so-called “Triangular osteosynthesis” (6, 7). Since then, lumbopelvic fixation and its variant, triangular fixation, became the gold standard in those cases where sacral fractures are associated to neurological deficits, persistent instability, vertical sacral fractures, lower bone quality or non-union (8–11).

The aim of this review is to analyze the available studies in the literature about lumbopelvic and triangular fixation, and assess clinical and radiological outcomes of the patients treated through those techniques, to better evaluate appropriate indications and impact on the natural history of the sacral fractures.

## Methods

### Study setting and design

**Figure 1:**
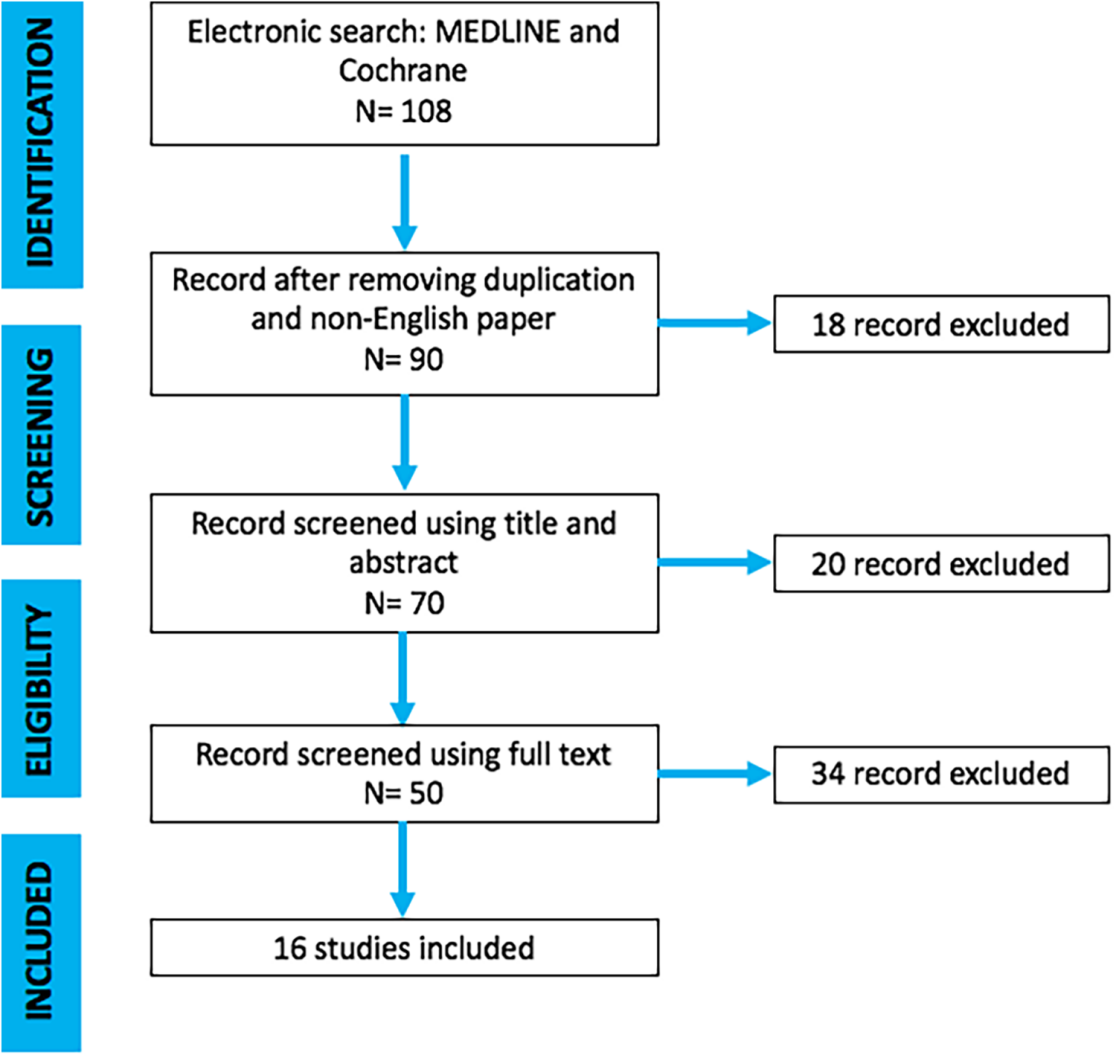
PRISMA flow-chart.

The present investigation represents a systematic literature review reported according to the Preferred Reporting Items for Systematic Reviews and Meta-Analyses (PRISMA) guidelines (Figure 1).

### Review questions

The review questions were formulated following the PICO scheme population (P), intervention (I), comparison (C), and outcome (O) as follows:

• Do patients suffering from posterior pelvic ring lesions report better clinical outcomes in term of complete healing rate (O), when treated through TOS (Triangular osteosynthesis) or LP (lumbopelvic) (I) in comparison to other techniques (C)?

### Inclusion and exclusion criteria

**Figure 2:**
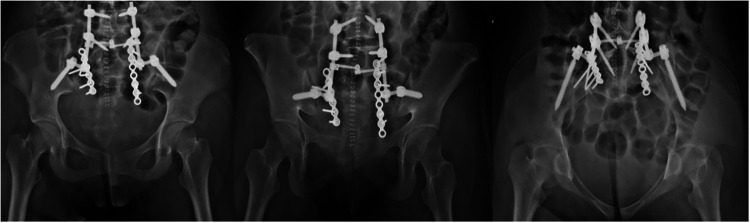
In our traumatology department, lumbopelvic fixation (LPF) involves placing pedicular screws in L5, sometimes L4, and bilaterally in the iliac bone, connecting them with rods on each side. The procedure can be performed in an open or closed manner, depending on the surgeon's skills and the patient's needs. To enrich LPF, a 6.5 or 7.3 cannulated screw can be added, positioned in the body of S1 or S2, in which case it is referred to as Triangular Osteosynthesis (TOS). Patients is placed prone on radiolucent surgical table, and the whole procedure is performed using intra-operative image intensifier.

In this review we considered the studies published as full-text articles in indexed journals, which investigated the value of TOS and LP for the management of sacral fracture (Figure 2). Only articles written in English with available abstract were included. No publication date limits were set. Surgical technique reports, expert opinions, letter to the editor, studies on animals, unpublished reports, cadaver or *in vitro* investigations, review of the literature, abstracts from scientific meetings and book chapters were excluded from the present review.

### Search strategy and study selection

Scopus, Cochrane Library, MEDLINE via PubMed and Embase were searched using the keywords: “vertical shear”, “pelvic ring fracture”, “Pelvic ring posterior fracture”, “vertically unstable pelvic injuries”, “lumbopelvic”, “spinopelvic”, “percutaneous lumbopelvic”, “triangular osteosynthesis” and their MeSH terms in any possible combination. The reference lists of relevant studies were screened to identify other studies of interest. The search was reiterated until December 31, 2022.

### Data extraction and analysis

Two independent reviewers collected the data from the included studies. Any discordances were solved by consensus with a third author. For each study included in the present analysis, the following data were extracted: Year, Types of Research Studies, demographic features, sex, age, diagnosis, previous hip surgery, pathogens, treatment performed, possible complications and outcomes, and follow-up. Numbers software (Apple Inc., Cupertino, CA) was used to tabulate the obtained data. Categorical variables are presented as frequency and percentages. Continuous variables are presented as means and standard deviation. Only one decimal digit was reported and was rounded up.

## Results

Descriptive data are given in (Figure 1). After screening 108 articles by title and abstract, 50 were considered eligible for the full-text analysis. 58 articles were excluded because they did not fulfill inclusion criteria. Finally, 16 studies (Tables 1–3) that met the inclusion criteria were included in this review (Figure 1). All these studies had a retrospective and case report descriptive design.

**Table 1:** Selected articles.

Studies	Number of patients	Sex		Age (year)	FU (months)
		M	F		
Mouhsine 2005	7	6	1	31	12
Schildhauer 2006	34	26	8	35	19
Gribnau 2009	8	–	–	29	36
Angthong 2010	1	1	–	26	21
Kell 2011	10	5	5	47	–
Soultanis 2011	1	1	–	19	30
Higgins 2012	1	–	1	74	24
Papakostidis 2015	1	1	–	16	24
Yu 2016	28	8	19	33.8	12
Sobhan 2016	14	11	3	37.9	32
Yano 2016	1	0	1	81	10
Jazini 2017	24	12	12	45	24
Sagi 2019	58	–	–	39	12
Korovesis 2020	22	15	7	36	61
Mathan Sakti 2020	1	1	–	28	–
Steelman 2021	1	–	1	25	21
Total	212	87	58	37.6688	24.14286

**Table 2:** Traumatic mechanism, classifications, sacral fracture and concomitant lesions.

Studies	Traumatic mechanism			Tile			Young-burgess				Denis			Sacral fractures morphology			Complications			
	Traffic accident	Fall from height	Other	A	B	C	APC	LC	VS	CM	I	II	III	H	U	Y	Visceral	Skeletal	Neurological	Uro-genital
Mouhsine 2005	1	5	1	–	–	4	–	–	–	–	–	–	–	3	–	–	–	8	4	–
Schildhauer 2006	–	–	35	–	–	34	–	–	–	–	11	30	2	–	–	–	–	–	22	–
Gribnau 2009	–	–	–	–	–	8	–	–	–	–	–	–	8	–	8	–	1	8	8	–
Angthong 2010	–	–	1	–	–	1	–	–	1	–	2	–	–	–	–	–	–	–	1	1
Kell 2011	3	–	–	–	–	10	–	–	–	–	–	–	–	–	–	–	–	–	–	–
Soultanis 2011	1	–	–	–	–	1	–	1	–	–	2	–	–	–	–	–	–	–	–	–
Higgins 2012	–	–	1	–	–	1	–	–	1	–	–	–	–	–	–	–	–	–	–	–
Papakostidis 2015	–	–	1	–	–	1	–	–	–	–	–	–	–	–	–	–	1	1	–	–
Yu 2016	13	15	–	–	–	26	–	–	10	16	6	7	15	–	–	–	13	26	2	–
Sobhan 2016	11	3	–	–	–	14	–	–	–	–	–	–	–	–	–	–	3	13	2	1
Yano 2016	1	–	–	–	–	1	1	–	–	–	1	–	–	–	–	–	–	19	–	–
Jazini 2017	13	6	5	–	–	–	6	5	2	–	4	7	13	4	1	2	6	5	–	–
Sagi 2019	–	–	–	–	–	58	–	–	58	–	–	–	–	–	–	–	–	8	4	9
Korovesis 2020	21	1	–	–	–	22	–	–	–	–	–	–	–	–	–	–	6	14	10	3
Mathan Sakti 2020	1	–	–	–	–	1	–	–	1	–	–	1	–	–	–	–	–	1	–	–
Steelman 2021	1	–	–	–	–	1	–	–	1	–	–	–	–	–	–	–	–	1	–	–
	66	30	44	–	–	183	7	6	74	16	30	45	38	7	9	2	30	104	53	14

**Table 3:** Treatment and complications.

Studies	Orthopedic treatment		Full weight bearing	Complications						Implant removal	Modified coleman methodology score	QoL questionnaires
	TOS	LP		Pain	Non-union/mal union	TVP/TEP	Neurological	Infection	Wound healing problems			
Mouhsine 2005	–	7	7 Early	–	2	–	0	1	1	7	50	
Schildhauer 2006	34	–	19 Early	–	–	1	7	2	1	15	64	
Gribnau 2009	4	–	6–12 months post-op	–	–	–	–	–	–	3	63	ISS
Angthong 2010	–	1	–	–	–	–	1	–	–	–	42	
Kell 2011	10	–	4 months post-op	–	2	–	5	–	–	5	55	ISS
Soultanis 2011	–	1	–	–	–	–	1	–	–	–	49	
Higgins 2012	–	1	–	1	–	–	–	–	–	–	40	
Papakostidis 2015	–	1	–	–	–	–	1	–	–	–	51	
Yu 2016	5	23	Delayed	21	–	–	–	1	2	–	57	HRQoL; EQ6D
Sobhan 2016	–	14	–	–	–	–	–	3	–	–	60	HRQoL; EQ6D
Yano 2016	–	1	8 weeks post–op	–	–	–	–	–	–	–	50	
Jazini 2017	–	24	–	–	–	–	–	2	–	11	64	
Sagi 2019	58	–	6 week post-op	56	3	–	5	3	5	15	50	SF–36
Korovesis 2020	–	–	8–10 weeks post–op	–	2	–	–	1	–	–	50	
Mathan Sakti 2020	–	1	–	–	–	–	–	–	–	–	55	
Steelman 2021	1	6	6 week post-op	1	–	–	–	–	–	1	51	
	112	80		83	9	1	19	13	9	57	53.19	

Overall, 212 patients (87 males, 58 females) suffering from pelvic fractures were collected. The mean age was 37.3 years. Mean follow-up, reported in all studies, was 24.7 months (Table 1). The causes of injury consisted in traffic accidents—most common mechanism of injury—(66 cases) followed by falls from height (30 cases) and others (44 cases) (Tables 2, 3).

The most common type of fracture was the Tile C reported in 183 patients (Table 2).

According to the Young-Burgess classification for pelvic ring injuries, 7 patients were identified as Anterior-Posterior Compression (APC), 6 with Lateral Compression (LC), 74 with Vertical Shear (VS) and 16 with Combined Mechanism (CM) (Table 2).

As regards sacral fractures, according to the Denis classification: 30 patients had a zone one I fracture; 45 had a zone II and 38 had a zone III (Table 2).

In terms of sacral fractures morphology, 7 were H-Type; 9 U-type; 2 Y-type (Table 2).

All patients had associated injuries (Table 2): 30 had also visceral lesions, among these 14 reported iliac artery bleeding, 6 severe pulmonary injuries. Among them were recorded: one bilateral pulmonary contusion, two unilateral pulmonary contusions, one bilateral pneumothorax, one unilateral pneumothorax, and one unilateral lung laceration. Other lesions were not specified. There were also 104 reported musculoskeletal associated injuries. The most common skeletal injury was lumbar lesion with 44 patients, and among these 3 had lumbar burst fractures, 2 were L5-S1 fracture dislocations, 39 had concomitant lumbar vertebral fractures and the others were not specified. Neurological associated injuries were reported in 53 patients, 4 with a pre-operative perineal neurological impairment, 4 had alterations of bladder and intestinal function, 3 developed sensorial impairment due to a complete cauda syndrome and 1 only partial cauda syndrome, 26 patients developed neurological non specified symptoms, 22 patients had non specified neurological deficit, 4 had lumbosacral plexus injuries and 10 patients had nerve root deficit; at last 14 patients reported bladder injuries (Table 2).

As regards the types of surgery, 112 were treated with TOS (Triangular Osteosynthesis), 101 underwent L5 to ilium fixation, 9 patients had also L4 involved in the fixation. 2 had fixation from L3 to L4 and ilium and for the other patients the treatment was not specified; 80 patients were treated with LP (Lumbopelvic) osteosynthesis, the other 23 patients were treated with other surgical techniques not relevant for this article (Table 3).

Weight bearing was described (Table 3) by many of the papers taken into account, and according to them an early weight bearing was achieved in 40 patients (21 treated with LP and 19 with TOS), between 30 days and 3 months a full weight bearing was reached by 64 patients (7 LP and 57 TOS). Ten patients (TOS) started full weight-bearing in 4 months, 4 patients in 6–12 months (12). Weight bearing was simply described as delayed in 28 patients (5 TOS and 23 LP), in 24 no weight bearing restrictions were reported (LP). Five studies do not report weight bearing data.

Post-operative complications were reported, especially infections and chronic pain (Table 3). The most common was pain due to the hardware, this was observed in 83 patients (62 treated with TOS and 21 with LP); non-union or malunion were observed in 9 patients; one patient reported TVP (13); 19 patients had neurological complications (among them, 2 drop foot, 2 radicular impingement, 1 cauda equina syndrome); 13 patients experienced wound infection (treated with debridement, antibiotics and in some cases with removal of the hardware) wound healing problems were reported in 9 cases.

In 57 patients implant removal was necessary to deal with the complications, 18 were treated with TOS and 44 with LP.

In 7 studies the authors used function questionnaires to evaluate outcome. Two studies used the injury severity score (ISS), 2 the Majed score, 2 the HRQoL (Health Related Quality of Life), 1 the SF36V2, 1 the SMFA and 1 the Matta criteria.

The Modified Coleman Methodology Score (mCMS) was used to evaluate the quality of studies, with a mean score for all studies of 53.18.

## Discussion

Our review confirms the heterogeneity of the data in the existing literature in terms of surgical management for unstable lumbosacral fractures (12–27).

Sacral fracture resulting in spinopelvic dissociation with neurological damage are high-energy injuries that occur rarely in polytrauma patients (The infrequency of these cases, the severity of the associated injuries and the absence of an accepted management flowchart make them highly morbid. If left untreated either intentionally or through misdiagnosis, progressive neurological dysfunction or painful deformity may occur (28, 29).

Misdiagnosis is frequent especially on plain radiographs, owing to the complexity of pelvic ring imaging, where the sacrum inclination and the overlaying bowel gas make the identification of the fracture very difficult. For this reason, multiplanar CT scan with 3D reconstructions is necessary for a correct and precise diagnosis of these unstable fractures and for the identification of associated injuries (13, 15, 30, 31).

Conservative treatment is discouraged while open reduction external fixation has proved to be a valid surgical option with good outcome (31–33).

The main role of surgery for the treatment of posterior pelvic ring lesions includes pelvic ring reconstruction, lumbopelvic stability restoration, fracture displacement prevention and correction to improve neurological deficiency. Even when treated correctly, with restoring of the spinopelvic stability and fracture consolidation, patients who have suffered this type of injury may develop sequelae from the injury itself or from the type of treatment. According to the literature, less than 50% of patients who have suffered complex sacral fractures return to their previous working conditions and functionality (34).

The 6 studies using TOS fixation (12, 13, 15, 20, 24) showed that this is a reliable form of fixation that allows early full weight-bearing while preventing loss of reduction and it's mostly recommended for comminuted vertical shear trans-foraminal sacral fractures (24). This surgical technique guarantees pelvic stability by combining indirect lumbopelvic fixation and direct screw fixation of the sacral fracture. Compared to direct fixation TOS may avoid excessive exposure, additional bleeding, and extra operation. However, some complications such as L5-S1 facet joint distraction with the need for a second surgery, and iatrogenic nerve injury, have been described in 3 of the 5 studies (12, 13, 24). Two studies (15, 27) did not report neither malunion nor nerve impairment after surgery. The main limitation of TOS technique is that it requires a highly skilled surgeon specialized in the treatment of pelvic ring fractures as it is a very complicated procedure (35, 36).

The 11 authors that used LP fixation (14, 16–26) showed that spinopelvic fixation is a good technique for sacral fractures with lumbopelvic dissociation, as it allows immediate mobilization, as well as weight bearing in the postoperative period (29). Pain, neurological impairment and infection rates were low, and mobilization was earlier. The benefits of minimally invasive LPF, however, may come with increased elective reoperations for removal of instrumentation. The main limitation of lumbopelvic fixation is that it cannot correct directly the sacral fracture leading in many cases to malunion or non -union.

In conclusion, lumbopelvic instable fractures include many severe injuries and are difficult to fix with a good outcome. LP and TOS have in many cases, showed satisfactory clinical outcomes in the treatment of LPF.

This study has some limitations. First, most of the studies included in the analysis were retrospective case series with no comparative group; unfortunately, no higher quality studies have been performed on the subject due to its high complexity; in fact, it is not possible to perform randomized clin- ical trials or double-blind controlled studies. Second, there is variability in age groups and also follow-up. Third, there is a lack of homogeneity in reporting fracture classification, evaluation scales, treatment, and outcomes.

## Conclusion

More accurate studies and stronger evidence are needed in order to address LP and TOS as gold standards in pelvic lesions involving posterior pelvic ring. However, actual findings in Literature suggest a good clinical and radiographic recovery through these surgical technique in the treatment of those fractures, especially when lumbar fracture are associated to the pelvic lesion, with a relative earlier weight bearing.

## Data Availability

The original contributions presented in the study are included in the article/Supplementary Material, further inquiries can be directed to the corresponding authors.
